# RSV-associated acute otitis media in children under five years old: a systematic review and meta-analysis

**DOI:** 10.7189/jogh.16.04202

**Published:** 2026-06-26

**Authors:** Rosa Hamilton-Smith, Sohail Ferdous, Daira Trusinska, Tsz-Yan Milly Lo, Ting Shi

**Affiliations:** Usher Institute, University of Edinburgh, Edinburgh, UK

## Abstract

**Background:**

Respiratory syncytial virus (RSV) is a leading cause of infant respiratory infections and an important contributor to acute otitis media (AOM) development. We aimed to synthesise evidence on RSV-associated AOM to support research to reduce its morbidity.

**Methods:**

In April 2025, we performed a systematic review and meta-analysis by searching the Embase, MEDLINE, and Global Health databases to identify studies reporting RSV-associated AOM in children aged 0–60 months. We extracted the proportions of RSV infections complicated by AOM, the proportions of AOM-associated samples with RSV detected, and the odds ratios for factors associated with AOM development in RSV-positive children.

**Results:**

Pooled estimates revealed 27.7% (95% confidence interval (CI) = 12.4–50.7) of RSV infections were complicated by AOM. RSV was detected in 23.6% (95% CI = 14.8–35.5) of AOM-associated samples, with detection rates slightly higher in nasopharyngeal samples 22.2% (95% CI = 13.2–34.8) than middle ear fluid samples 18.8% (95% CI = 6.6–42.9). Bacterial co-infections were present in 70.1% (95% CI = 43.4–87.7) of RSV-positive middle ear fluid samples among children with AOM. Factors associated with AOM development in RSV-positive children included high viral load, *Streptococcus pneumoniae* co-infection, being aged between 3 and 12 months, and fever.

**Conclusions:**

RSV is a prominent viral contributor to AOM pathogenesis; however, significant heterogeneity and methodological differences across studies limit the accuracy and generalisability of findings. Standardised prospective studies are needed to further investigate risk stratification and evaluate the effectiveness of interventions to prevent RSV-associated AOM and improve its management.

**Registration:**

PROSPERO: CRD42025642807.

Respiratory syncytial virus (RSV) infection is a leading cause of mortality and hospitalisation in children aged <5 years, accounting for an estimated 3.6 million hospitalisations and 101 400 deaths globally in 2019 [1]. In children, it is responsible for significant morbidity, causing complications such as bronchiolitis and acute otitis media (AOM) [2].

AOM refers to an acute inflammatory response in the middle ear to bacterial/viral/co-infection, commonly manifesting as bulging and erythema of the tympanic membrane [3,4]. Other symptoms of AOM include otalgia, fever, and conductive hearing loss. Long-term complications include tympanic membrane perforation and chronic middle ear infection, which may lead to delays in speech/language development or permanent hearing loss [5]. AOM imposes a substantial paediatric health burden, affecting up to 75% of children before the age of five years old [6]. It is also one of the most common paediatric diagnoses in the emergency department [7].

Studies have often linked RSV to AOM by detecting RSV in nasopharyngeal (NP) and middle ear fluid (MEF) samples of children with AOM [2,8,9]. RSV-induced inflammation can impair eustachian tube function, leading to a negative pressure buildup in the middle ear. Pressure changes facilitate pathogen migration from the nasopharynx, potentially leading to AOM [10]. RSV is not established as directly causal of AOM, as its development is multifactorial and often involves multiple pathogens [11]. However, it is a recognised facilitator of AOM pathogenesis by promoting inflammation and middle ear dysfunction [12].

Research on RSV-associated AOM is limited. To our knowledge, only one systematic review has been published on this topic, which calculated the proportion of RSV in children with AOM and assessed the prevalence of bacterial co-infections in RSV-positive AOM cases [10]. Our review extends current research by differentiating between and performing analyses for two distinct clinical pathways: children with RSV infections which are then complicated by AOM, and children already diagnosed with AOM who have RSV detected within samples. Additionally, we provide separate analyses for RSV occurrence in NP *vs.* MEF samples, as sample types may differ in diagnostic performance. Finally, we provide a narrative synthesis of odds ratios (ORs) to identify risk factors for AOM development in children with RSV infection. We hypothesised that AOM frequently complicates RSV infections and that RSV is commonly detected in samples from children diagnosed with AOM.

Establishing a better understanding of the epidemiological impact of RSV-associated AOM is essential to inform health policies, target clinical interventions, and develop vaccination strategies.

## METHODS

### Definitions of terms

The time window denoted the maximum interval during which participants with RSV infections were monitored for AOM development. Record-based studies were those that utilised existing medical databases or clinical charts to retrospectively identify AOM occurrences in RSV-infected children. Active surveillance refers to studies that prospectively followed a cohort of RSV-infected children to systematically screen for AOM. AOM-associated samples are samples taken from children with a diagnosis of acute otitis media.

### Search and screening

In this review, we followed the PRISMA guidelines [13]. An initial project proposal was registered with PROSPERO (registration number: CRD42025642807). On 10 April 2025, we conducted a search in Embase, MEDLINE, and Global Health to identify studies published between 1 January 2004 and 1 January 2025. The search was restricted to this 21-year window to capture data relevant to the era of modern molecular diagnostics and contemporary RSV surveillance practices. The search strategy incorporated relevant keywords such as RSV, otitis media, bronchiolitis and infants (Materials S1 and S2 in the **Online Supplementary Document**).

Inclusion criteria encompassed English-language studies involving children aged <5 years with confirmed diagnoses of both RSV and AOM. Eligible studies reported proportions of RSV-associated AOM cases and/or ORs examining this association. Studies were excluded if they did not meet these criteria, were abstract-only, were literature reviews, were not in English, or were published outside the specified date range. Two independent reviewers screened titles, abstracts, and full texts using Covidence (Veritas Health Innovation, Melbourne, Victoria, Australia) [14]. Conflicts during screening were resolved through consensus amongst reviewers.

### Data extraction

Data were extracted independently by two reviewers (RHS and SF) using pre-specified data extraction forms. An analysis plan to guide data extraction was developed prior to the review process (Figure S1 in the **Online Supplementary Document**). Key variables included study design, country, population demographics, sample size, care setting, and participants’ age range. We then extracted proportions of RSV infections complicated by AOM and proportions of samples taken from AOM-diagnosed children that were RSV-positive. To maintain the independence of subsets, studies were categorised by their baseline populations: studies observing complications amongst children with respiratory infections (including RSV) informed the first estimate, whilst studies enrolling children with existing AOM diagnoses informed the latter estimate.

Additionally, we extracted both adjusted and unadjusted ORs for risk factors associated with the likelihood of AOM development in children with RSV infections. Where available, univariable ORs were manually calculated by the reviewers (Material S3 in the **Online Supplementary Document**).

We conducted quality analysis using the JBI critical appraisal tools [15]. Following the approach used in recent systematic reviews on RSV, we categorised studies according to their risk-of-bias scores, with scores >75% considered low risk of bias, 50–75% considered moderate risk, and <50% considered high risk of bias [16,17].

### Statistical analysis

We calculated two primary outcomes. First, the proportion of RSV infections complicated by AOM, and second, the proportion of AOM-associated samples with RSV detected, using random-effects modelling with logit-transformed proportions (Material S4 in the **Online Supplementary Document**). To handle zero-event cells, a constant continuity correction of 0.5 was applied to all studies. Studies in the latter proportion were grouped by sample type (NP or MEF), and the pooled proportion of bacterial co-infections among RSV-positive MEF samples was also calculated. Meta-analysis was conducted only when three or more studies provided data. We calculated 95% confidence intervals (CIs) and assessed heterogeneity using the *I^2^* statistic, with values of <25%, 25–50%, and >50% indicating low, moderate, and high heterogeneity, respectively. To account for the variability in future studies, prediction intervals were also calculated.

Univariate meta-regression and subgroup analyses with random-effects modelling were performed for both proportions to explore potential sources of heterogeneity. For meta-regression, continuous moderators included publication year, maximum participant age, and the time window between RSV infection and AOM diagnosis (the latter is applicable only to the first proportion). Categorical moderators included study design (active surveillance *vs.* record-based), setting (outpatient *vs.* hospital), RSV detection method (polymerase chain reaction (PCR) *vs.* assay), and AOM-identification method (otoscopy *vs.* International Classification of Diseases, Ninth Revision (ICD-9) codes). Subgroup analyses were also performed for study design, setting, RSV detection method, time-windows (≤14 days *vs.* >14 days) and AOM identification method. Differences between subgroups were formally assessed using χ^2^ tests in the random-effects model. For categorical regression and subgroup analyses, a minimum of three studies per stratum was enforced to maintain model stability. For all analyses above, a *P*-value <0.05 was considered evidence of statistical significance.

Further sensitivity analyses were performed to evaluate the robustness of proportions. For the first proportion, two sensitivity analyses were conducted: one excluding studies that did not report time windows, and the other excluding studies that did not report AOM-identification methods. For the second proportion, a sensitivity analysis was performed by excluding studies that used non-PCR-based techniques to detect RSV. For both proportions, sensitivity analyses excluding studies which received JBI scores <75% were performed to assess the impact on pooled proportions. Additionally, leave-one-out sensitivity analyses were conducted to assess the robustness of proportions. Funnel plots and Egger’s test, along with trim-and-fill analyses, were also used to assess the risk of publication bias when a minimum of 10 studies were available. We performed a meta-analysis using the ‘meta’ package in *R*, version 4.2.3 (R Core Team, Vienna, Austria) [18].

## RESULTS

**Figure 1:**
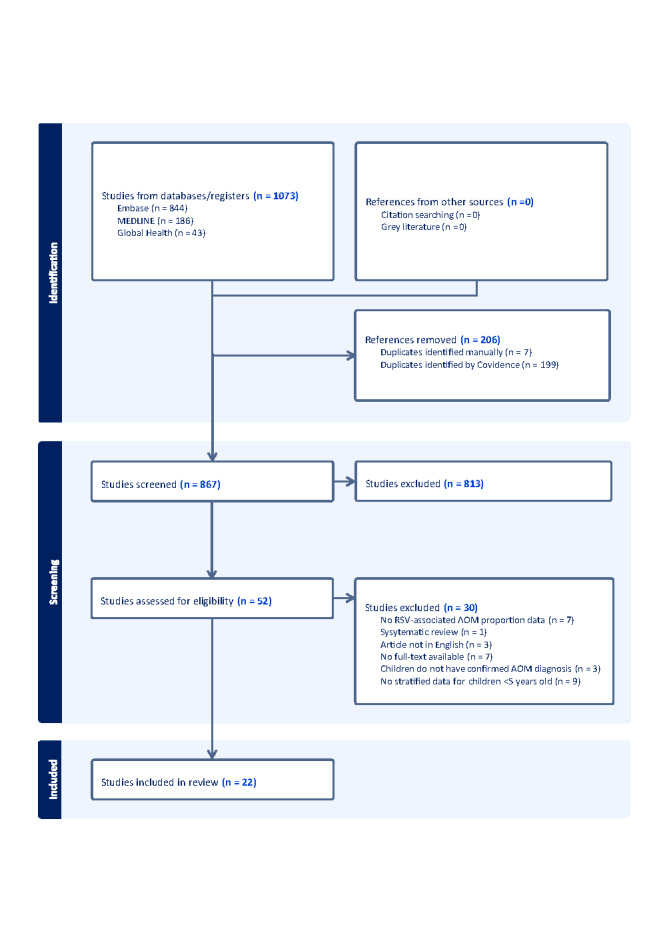
PRISMA flowchart showing the study screening process. AOM – acute otitis media, RSV – respiratory syncytial virus.

We identified 1073 studies from the initial search. After screening, a total of 22 studies were included in the analysis (**Figure 1**) [11,19–39]. Of the studies included, 13 (59.0%) were prospective, seven (33.3%) were cross-sectional, one (4.5%) was a time-series analysis, and one (4.5%) was a retrospective case-control. Studies contained data from hospital or outpatient settings, with 15 (68.1%) conducted in secondary care, five (22.7%) in primary care, and two (9.1%) in tertiary care. 20 (90.9%) of studies were conducted in high-income countries (HICs), with one (4.5%) study from an upper-middle-income country and one (4.5%) from a lower-middle-income country (LMIC).

Based on the risk of bias assessment, seven (31.8%) studies were classified as having a low risk of bias (JBI score >75%) and 15 (68.2%) as having a moderate risk of bias (JBI score 50–75%) (Tables S1 and S2, and Material S5 in the **Online Supplementary Document**).

### Proportion of RSV infections complicated by AOM

**Figure 2:**
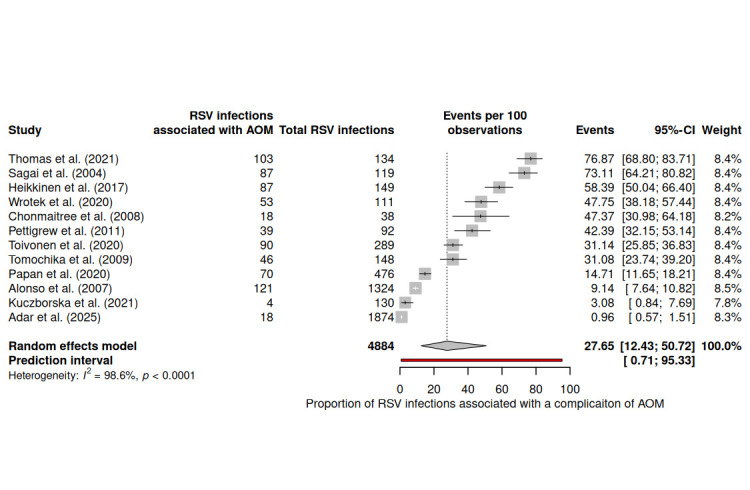
Proportion of RSV infections complicated by AOM. Forest plot showing that across 12 studies, the pooled proportion of RSV infections complicated by AOM was 27.7% (95% CI = 12.4–50.7). AOM – acute otitis media, RSV – respiratory syncytial virus.

Across 12 studies, 27.7% of RSV infections were complicated by AOM (95% CI = 12.4–50.7, *I^2^* = 98.6, prediction interval = 0.71–95.3) (**Figure 2**).

### Proportion of AOM-associated samples with RSV detected

**Figure 3:**
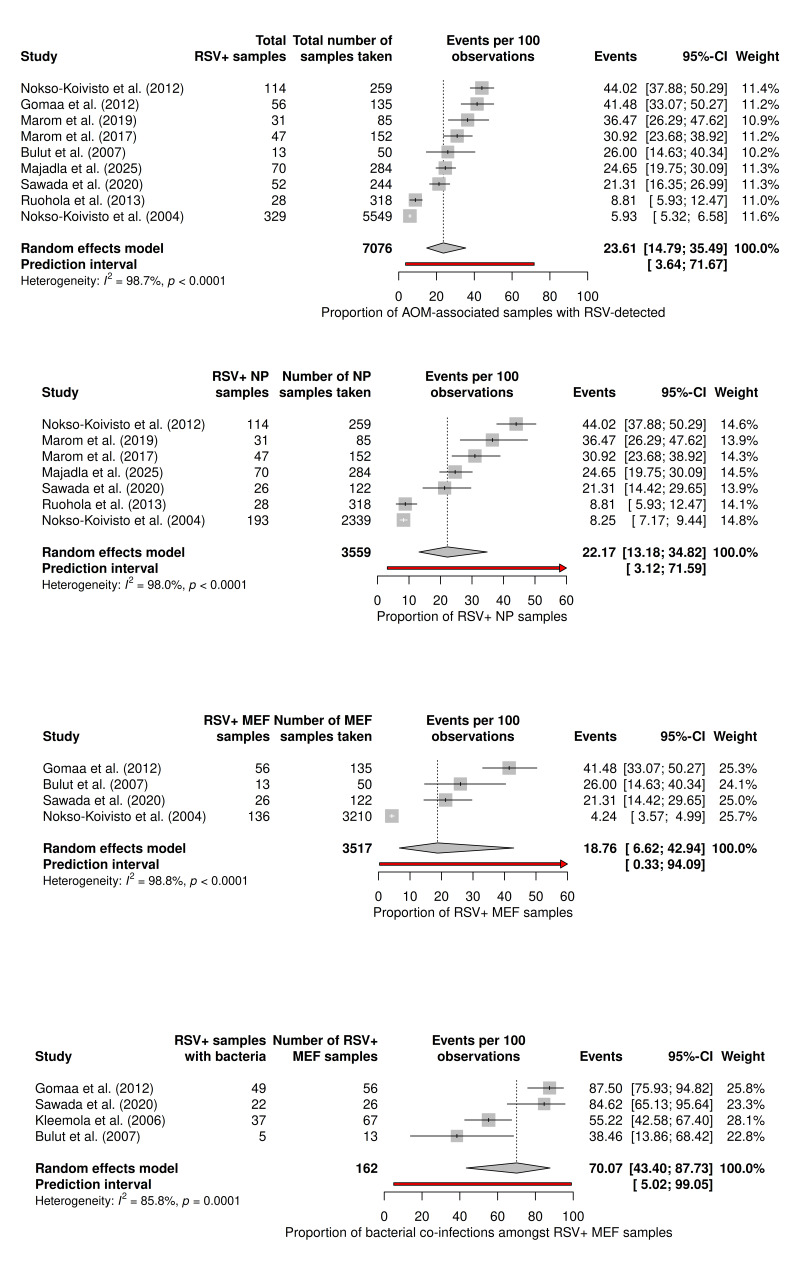
Proportion of AOM-associated samples with RSV detected. **Panel A.** 23.6% (95% CI = 14.8–35.5) of AOM-associated samples were RSV+. **Panel B.** 22.2% (95% CI = 13.2–34.8) of NP samples were RSV+. **Panel C.** 18.8% (95% CI = 6.6–42.9) of MEF samples were RSV+. **Panel D.** 70.1% (95% CI = 43.4–87.7) of RSV+ MEF samples contain bacterial coinfections. AOM – acute otitis media, MEF – middle ear fluid, NP – nasopharyngeal, RSV+ – tested positive for respiratory syncytial virus.

Across nine studies, 23.6% of AOM-associated samples tested positive for RSV (95% CI = 14.8–35.5, *I^2^* = 98.9, prediction interval = 3.6–71.7). When stratified by sample type, 22.2% (95% CI = 13.2–34.8, *I^2^* = 98.0) of NP samples were RSV-positive. The proportion of RSV-positive MEF samples was lower at 18.8% (95% CI = 6.6–42.9, *I^2^* = 98.8). The proportion of bacterial co-infections among RSV-positive MEF samples was 70.1% (95% CI = 43.4–87.7, *I^2^* = 85.8) (**Figure 3**, Panels A–D).

### Risk factors for AOM development in children with RSV infections

Among studies reporting risk-factor-based ORs, only Pettigrew *et al.* adjusted for AOM-related covariates, including age, ethnicity, daycare attendance, breastfeeding status, tobacco smoke exposure, antibiotic use, and the presence of concurrent pathogens in specimens. The remaining ORs are unadjusted.

Tomochika *et al.* reported that fever (≥37.5°C) was significantly associated with risk of AOM in RSV-positive children (OR = 2.3; 95% CI = 1.5–30.0) [25]. Pettigrew *et al.* found risk of AOM was associated with high RSV viral load (>3.16 × 10^7^ copies/ml) (OR = 2.6; 95% CI = 1.3–5.2), and further increased when *Streptococcus pneumoniae* co-infection was present (OR = 4.4; 95% CI = 1.9–10.2) [11]. Three studies provided data on age as a risk factor for AOM development in children infected with RSV. Wrotek *et al.* reported RSV-positive children aged over three months were almost 10 times more likely to develop AOM compared to their younger counterparts (OR = 9.8; 95% CI = 3.6–26.7) [22].

Self-calculated ORs revealed RSV-positive children ≤12 months were at significantly higher risk of suffering from AOM compared to children aged >12–72 months (OR = 5.3; 95% CI = 2.9–9.7) [20]. In another study, RSV-positive children aged <12 months were at greater risk of AOM than children in the 12–24-month age group, though the *P* value did not reach significance (OR = 7.9; 95% CI = 1.0–63.6) (**Table 1**) [21].

**Table 1:** Odds of RSV-positive children with AOM having a specific risk factor/symptom in comparison to RSV-positive children without AOM

Study	RSV-positive children without AOM, n	RSV-positive children with AOM, n	Reported ORs for risk factors/symptoms in RSV-positive children with AOM *vs.* RSV-positive children without AOM*	Calculated ORs for risk factors/symptoms in RSV-positive children with AOM *vs.* RSV-positive children without AOM†
Tomochika *et al.*, 2009 [25]‡	46	102	Fever (temperature ≥37.5°C): OR = 2.26 (95% CI = 1.53–30.01, *P* = 0.005)	NA
Pettigrew *et al.*, 2011 [11]	53	39	High viral (RSV) load (>3.16 × 10^7^ copies/ml): OR = 2.60 (95% CI = 1.30–5.20); High viral (RSV) load and *Streptococcus pneumoniae* presence: OR = 4.40 (95% CI = 1.9–10.19)§	NA
Wrotek *et al.*, 2020 [22]	58	53	Aged >3 months: OR = 9.8 (95% CI = 3.6–26.7)§	NA
Sagai *et al.*, 2004 [20]	110	120	NA	Aged ≤12 months compared to being aged >12–72 months: OR = 5.3 (95% CI = 2.9–9.7, *P* < 0.0001)
Heikkinen *et al.*, 2017 [21]	150	148	NA	Aged <12 months compared to being aged 12–24 months: OR = 7.9 (95% CI = 1.0–63.6, *P* = 0.05)

AOM – acute otitis media, CI – confidence interval, NA – not available, OR – odds ratio, RSV– respiratory syncytial virus

*Reported means the OR was extracted directly from the paper.

†Calculated means the OR was manually derived and calculated from raw study data.

‡OR was adjusted for age, ethnicity, daycare attendance, breastfeeding, tobacco exposure, and antibiotic use.

§*P*-value was not available; however, studies reported ORs as statistically significant.

### Univariate meta-regression and subgroup analyses

Univariate meta-regression found that study design (record-based *vs.* active surveillance) was the only tested variable that significantly affected the proportion of RSV infections complicated by AOM (*P* < 0.0001). Subgroup analyses by study design showed similar results: 54.4% (95% CI = 41.0–67.2, *I^2^* = 94.2) of RSV infections were complicated by AOM in active-surveillance studies, compared with only 7.1% (95% CI = 2.2–22.3, *I^2^* = 97.4) in record-based studies. Further subgroup analyses revealed that proportions were higher in outpatient studies, 53.9% (95% CI = 33.7–73.0, *I^2^* = 98.6), compared to hospital-based studies, 17.9% (95% CI = 5.6–44.1, *I^2^* = 96.1). Studies utilising PCR-based techniques reported a higher proportion of RSV infections complicated by AOM 32% (95% CI = 11.9–61.3, *I^2^* = 98.6) compared to those using only assay-based methods 20.5% (95% CI = 3.8–62.5, *I^2^* = 98.8), but the test for subgroup differences revealed this difference was not statistically significant (*P* = 0.6). Subgroup differences between studies that monitored children with RSV infections for >14 days and those for ≤14 days were not significant.

Regarding the proportion of AOM-associated samples with RSV detected, both meta-regression and subgroup analyses revealed no significant sources of heterogeneity or differences between subgroups (Figure S2 and S3, Tables S3 and S4 in the **Online Supplementary Document**).

### Sensitivity analyses

Sensitivity analyses for the proportion of RSV infections complicated by AOM revealed that when studies using non-otoscopy-based techniques to identify AOM-cases and studies that did not report time-window data were excluded, the pooled proportion significantly increased from 27.7–51.3% (95% CI = 38.4–64.0, *I^2^* = 94.2) and 54.4% (95% CI = 41.0–67.2, *I^2^* = 94.2), respectively.

Regarding the proportion of AOM-associated samples with RSV detected, excluding studies that used non-PCR-based RSV detection methods did not significantly alter this proportion. Sensitivity analyses excluding studies with a JBI score <75% were conducted for both proportions. Neither sensitivity analysis had a significant effect on overall pooled proportions or *I^2^* values (Figures S4–8 in the **Online Supplementary Document**).

A leave-one-out sensitivity analysis was performed for both primary outcomes. For the proportion of RSV infections complicated by AOM, the pooled estimate ranged from 23.8% (95% CI = 10.4–46.0) to 35.0% (95% CI = 19.4–55.6) when any single study was excluded. Regarding RSV detection in AOM-associated samples, the pooled estimate ranged from 21.5% (95% CI = 13.1–33.3) to 27.6% (95% CI = 19.3–37.8) (Figures S9 and S10 in the **Online Supplementary Document**).

### Publication bias

For the proportion of RSV infections complicated by AOM, visual inspection of the funnel plot suggested possible asymmetry; however, Egger’s test did not provide statistical evidence of publication bias (*P* = 0.4). Trim-and-fill analysis imputed two potentially missing studies, reducing the pooled proportion from 27.7% (95% CI = 12.4–50.7) to 20.1% (95% CI = 8.4–40.9) (Figures S11 and S12 in the **Online Supplementary Document**).

For the proportion of AOM-associated samples with RSV detected, the funnel plot demonstrated clear asymmetry, which was supported by Egger’s test (*P* = 0.02). Trim-and-fill analysis imputed five potentially missing studies, leading to a substantial reduction in the pooled estimate from 23.6% (95% CI = 14.8–35.5) to 9.3% (95% CI = 4.3–18.9) (Figures S13 and S14 in the **Online Supplementary Document**).

## DISCUSSION

In this study, we quantified the association between RSV and AOM in children aged <5 years, whilst also providing insight into patterns of co-infection and risk factors related to RSV-associated AOM development. 27.7% of RSV infections resulted in complicating AOM. Subgroup analyses showed higher proportions of RSV infections complicated by AOM in active-surveillance studies (54.3%) than in record-based studies (7.1%), with meta-regression confirming study design as a significant source of heterogeneity (*P* < 0.05).

RSV presence was detected in 23.6% of AOM-associated samples. When grouped by sample type, RSV infection was detected in 22.2% of NP and 18.8% of MEF samples from AOM-diagnosed children. Bacterial co-infections were detected in 70.1% of RSV-positive MEF samples.

### RSV infections complicated by AOM

Over one-quarter of RSV infections were complicated by AOM. However, this proportion exhibited significant heterogeneity (*I^2^* = 98.6), indicating that this estimate represents a broad descriptive aggregate rather than a stable epidemiological estimate. This variance is likely due to methodological differences, with univariate meta regression analyses identifying study design as a primary driver of heterogeneity. This finding was supported by subgroup analyses revealing that AOM occurrence was significantly more common in studies with active-surveillance designs (54.3%) than in record-based designs (7.1%). Record-based studies did not track children's development of AOM over time and relied on existing medical records, which may lead to underreporting given AOM’s transient nature. This suggests that in routine clinical practice, AOM is frequently overlooked or under-documented.

Additionally, RSV-associated AOM occurrence was observed more commonly in outpatient studies than hospital-based (53.9% *vs.* 17.9%). This likely reflects differences in study design rather than true subgroup differences, as most hospital-based studies were record-based and typically focused on reporting more severe complications such as bronchiolitis, whereas outpatient studies all had active surveillance designs.

The influence of study design was further evidenced by sensitivity analyses. The pooled proportion of RSV infections complicated by AOM rose significantly when excluding studies that failed to report time windows (54.4%) and when excluding studies that relied on diagnostic coding rather than clinical otoscopy (51.3%). In both analyses, the majority of excluded studies were record-based. Collectively, these findings suggest prospective active-surveillance frameworks are essential for accurately determining the proportion of RSV infections complicated by AOM.

### AOM-associated samples with RSV detected

We found that 23.6% (95% CI = 14.8–35.5, *I^2^* = 98.9) of AOM-associated samples were RSV-positive. The existing systematic review by Kenmoe *et al.* reported that the proportion of RSV among children with AOM was lower at 16.9% (95% CI = 11.0–23.8, *I^2^* = 94.9) [10]. It should be noted that the above proportions are not directly comparable, as Kenmoe *et al.* calculated a case-based proportion, whereas ours was sample-based.

Several other factors may explain the differences in the estimates above. Kenmoe *et al.* included studies from Chinese databases and did not impose a publication date restriction. The resulting broader scope and the larger number of studies included could enhance the precision of their estimated proportion. However, the inclusion of older studies could lower RSV-positivity rates, as they may rely on less accurate non-PCR-based diagnostic techniques, thereby reducing reported proportions [40]. Our sensitivity analysis excluding non-PCR-based studies did not significantly affect our pooled estimate, although only two studies in our proportion did not utilise PCR-based techniques (Figure S6 in the **Online Supplementary Document**).

Subgroup analyses by Kenmoe *et al.* found that the RSV proportion among AOM-diagnosed children was highest in inpatient studies, among children aged 0–11 months, in studies that performed both MEF and NP testing, and in seasonal studies. We could not assess these subgroups due to insufficient data. Our own subgroup analyses showed no significant differences between prospective and cross-sectional studies, and between studies that identified AOM cases via otoscopy and those that used ICD-9 codes. This was supported by meta-regression, which found that none of the tested variables had a statistically significant effect on proportions.

Our analysis found that RSV was present in 22.2% of NP samples and 18.8% of MEF samples. These differences likely reflect both biological compartmentalisation and differences in sampling practices. NP sampling may be less accurate than MEF, as NP samples detect pathogens in the upper airway that may not contribute to AOM pathogenesis [41]. However, unlike NP aspiration, MEF aspiration requires clinicians to perform tympanocentesis, an invasive procedure with associated risks including tympanic membrane perforation [42]. As tympanocentesis is generally performed in more complex AOM cases, MEF proportions are not a random subset of all AOM-diagnosed children, and this selection bias must be considered when interpreting aetiology [43]. Future studies should balance diagnostic specificity with procedural risk when selecting sampling methods.

### Bacterial co-infections

We found that 70.0% of RSV-positive MEF samples had bacterial co-infections. Similarly, Kenmoe *et al.* found RSV-bacterial co-infections in 67.4% of RSV-associated AOM cases and reported that *Streptococcus pneumoniae* was the bacterium most commonly co-detected with RSV, followed by *Haemophilus influenzae* [10]. Previous studies have demonstrated that RSV can promote bacterial colonisation and middle ear infection by disrupting the nasopharyngeal flora, impairing mucociliary clearance, and increasing bacterial adherence to epithelial cells [44,45]. RSV-bacterial coinfections can both increase the risk of AOM development and prolong its duration by amplifying inflammatory responses in the nasopharynx and impairing antibiotic penetration [46,47].

Supporting this, Pettigrew *et al.* reported that infants with high RSV viral load combined with *Streptococcus pneumoniae* presence were four times more likely to develop AOM compared to infants with neither factor present 4.40 (95% CI = 1.90–10.19) [11]. Therefore, clinically identifying patients with these co-infections is important, as it may influence the course, severity and treatment of AOM.

It should be noted that RSV-bacterial co-infection dynamics could have been influenced by the widespread implementation of pneumococcal conjugate vaccine (PCV) in the early 2000s [48]. Following PCV implementation, there was a shift in AOM aetiology, with vaccine-covered serotypes declining while non-covered serotypes and organisms such as non-typeable *Haemophilus influenzae* became more predominant [49]. Evidence is inconclusive regarding PCV’s impact on all-cause AOM; its effect on virus-associated AOM remains unclear [50].

### Risk factors linked to RSV-associated AOM

Evidence suggests that age is a significant risk factor for AOM. Sagai *et al.* reported RSV-infected children ≤12 months had a 5-fold increased risk of developing AOM (OR = 5.3; 95% CI = 2.9–9.7), while Heikkinen *et al.* reported an even higher risk for those <12 months (OR = 7.9; 95% CI = 1.0–63.6) [20,21]. This aligns with subgroup analyses from Kenmoe *et al.* showing RSV-associated AOM proportions were highest among children aged 0–11-month old [10]. However, Wrotek *et al.* reported that RSV-infected children aged >3 months were at greater risk of AOM (OR = 9.8; 95% CI = 3.6–26.7) [22]. Young children are anatomically more susceptible to AOM due to shorter horizontal eustachian tubes, which promote efficient transfer of pathogens from the nasopharynx to the middle ear [51]. Young children’s immature immune responses also impair their ability to clear RSV, raising infection risk [52]. However, children aged <3 months may have reduced AOM incidence due to protective factors, including passive immunity from maternal antibodies and more limited exposure to communal environments [23,53]. Overall, this evidence suggests AOM interventions and detection strategies should be prioritised for children within the first year of life.

In addition to age-specific risk factors, Pettigrew *et al.* reported that RSV-positive children with high viral loads were more than twice as likely to develop AOM as those with medium or low viral loads (OR = 2.60; 95% CI = 1.30–5.20) [11]. However, a 2015 cohort study in Japan of 362 children aged ≤1 year reported that whilst viral load was associated with symptomatic RSV infection, it was not significantly associated with the development of AOM [54]. Fever was the only symptom-based OR reported in the literature, with Tomochika *et al.* finding that RSV-associated AOM was more common in children with fever (OR = 2.26; 95% CI = 1.53–30.01) [25]. However, because fever is a fairly nonspecific symptom, it is unlikely to be a useful indicator of RSV-associated AOM.

Reporting of AOM-related risk factors across the literature is scarce, and the existing ORs mentioned above are imprecise, as evidenced by wide confidence intervals. To improve diagnostic precision, further research is needed, and the use of multimodal scoring systems that combine clinical and demographic data could provide a more effective framework for identifying high-risk infants.

### Prevention and management

The multifaceted role of RSV in AOM development, emphasised by the data above, has implications for infection management. Although several studies in this review reported RSV-bacterial co-infection occurrence as relatively common, antibiotics are often unnecessary in mild bacterial and/or viral AOM cases, with an estimated 80% of cases resolving without antibiotics, and complications remaining rare regardless of treatment status [55,56]. Despite this, prescribing rates for AOM reach up to 95% in high-income nations such as the USA, Canada, and Australia [57]. Elevated rates of antimicrobial prescription are associated with increased antimicrobial resistance and intestinal dysbiosis, which can impair paediatric health and development [58,59]. Additionally, a UK study in 2023 observed rising rates of antimicrobial resistance amongst key AOM-associated pathogens, such as *Haemophilus influenzae*, which could increase the risk of treatment failure [60]. This evidence underscores the limitations of antibiotic-focused strategies, and draws attention to a need for vaccination and antibody-based strategies that target pathogenic AOM contributors.

Previous vaccination strategies have already proven effective at mitigating AOM. Randomised-controlled trials have found live-attenuated trivalent influenza vaccines capable of reducing influenza-associated AOM cases by 83% compared to placebo, and pneumococcal conjugate vaccines have been reported to decrease vaccine-serotype AOM cases by an estimated 57% and all AOM episodes by 6% [61,62]. In parallel, recently introduced maternal RSV vaccines could become a promising AOM intervention. While this vaccine has shown efficacy in reducing severe RSV disease in newborns, its impact on AOM development remains unclear [63].

Antibody-based RSV treatments have yielded more mixed results. Whilst one study reported that children who received high-dose RSV immunoglobulin (RSVIG) had significantly fewer AOM episodes than those who received no RSVIG (0.15 *vs.* 0.78 episodes per season; *P* = 0.003), this effect was non-specific, as infants also received antibodies targeting other pathogens [64]. Palivizumab, which targets the RSV fusion (F) protein, doesn’t reduce the incidence of otitis media [65]. In contrast, the newly introduced monoclonal antibody nirsevimab, which targets the more immunogenic prefusion form of the F protein, offers passive immunity against severe RSV disease and may therefore reduce AOM incidence [66,67]. Further studies are needed to clarify the impact of maternal vaccination and nirsevimab on the occurrence of AOM.

### Limitations

In this meta-analysis, we quantified the association between RSV infection and AOM in children by synthesising the proportions of RSV infections associated with AOM, AOM-associated samples with RSV detected, and odds ratios to provide a comprehensive overview of their relationship. However, our review has several limitations. A major limitation of our study is the significant heterogeneity in our estimates, as reflected in high *I^2^* values and broad prediction intervals. This is likely attributable to variations in methodology across studies. Consequently, pooled proportions should be interpreted as descriptive aggregates of current literature rather than definitive, universal population estimates.

The inclusion of both retrospective and prospective studies in this analysis introduces a major source of bias and heterogeneity. Retrospective studies, which rely on preexisting medical records, are subject to underreporting and missing data, and may therefore underestimate the occurrence of RSV-associated AOM. Additionally, many record-based studies did not provide time-window data, which may have led to mixing incident AOM cases with prevalent or ongoing AOM, as well as unrelated RSV carriage or detection. Finally, population demographics and study characteristics were reported only sparsely across studies, limiting our ability to control for confounders and perform subgroup analyses. Caution is thus warranted when interpreting these highly heterogeneous estimates.

Regarding publication bias, inspection of funnel plots, Egger’s test, and trim-and-fill analyses suggested potential small-study effects for the pooled proportion of AOM-associated samples with RSV detected. This suggests that the proportion of RSV in AOM-associated samples may be inflated because few studies report lower proportions. However, these results should be interpreted with caution, given the high heterogeneity and the limited number of included studies – both of which are known to affect the reliability of publication bias tests [68].

Finally, all but two of the included studies were conducted in HICs, limiting the applicability of our results to global populations. Whilst antimicrobial use for AOM is substantial in HICs and a driver of antimicrobial resistance, 99% of the estimated 3.2–3.6 million annual RSV hospitalisations occur in LMICs, where otitis media is also more prevalent [69–71]. To address this disparity, there is a critical need for studies examining the epidemiology of AOM and RSV in LMIC settings.

## CONCLUSIONS

In this review, we observed that notable proportions of RSV infections are complicated by AOM, and that RSV is often detected in AOM-associated samples, frequently alongside bacterial co- infections. However, significant heterogeneity, methodological variations and limited geographical representation limit the reliability and generalisability of these findings. Future prospective cohort studies are needed to clarify this association and identify relevant risk factors. Given the substantial health burden of AOM and concerns surrounding antibiotic overuse, emerging RSV interventions should be evaluated for their potential to reduce AOM incidence.

## Additional material


Online Supplementary Document
