# Supplementary material for: RSV-associated acute otitis media in children under five years old: a systematic review and meta-analysis
Source: J Glob Health. 2026 Jun 26;16:04202. doi: 10.7189/jogh.16.04202 (PMC13307545; doi:10.7189/jogh.16.04202)
Supplement: Online Supplementary Document [file jogh-16-04202-s001.pdf]

Supplement to: Hamilton-Smith R, Ferdous S, Trusinska D, Lo TYM, Shi T. RSV-associated acute otitis media in children under five years old: a systematic review and meta-analysis. J Glob Health. 2026;16:04202.

Online supplementary document

Contents

Material S1: PRISMA checklist ... 2

Material S2: Search strategy ... 5

Figure S1: Analysis plan ... 6

Material S3: Proportion calculations ... 7

Material S4: Manual odds ratio calculation ... 7

Material S5: Quality assessment ... 8

Table S1: Study design and population demographics ... 10

Table S2: Surveillance time-window data ... 14

Table S3: Meta Regression: The proportion of RSV infections complicated by AOM ... 14

Table S4: Meta Regression: The proportion of AOM-associated samples with RSV-detected ... 15

Figure S2: Subgroups for the proportion of RSV infections complicated by AOM ... 15

Figure S3: Subgroups for the proportion of AOM-associated samples with RSV detected ... 15

Figure S4: Sensitivity analysis for the proportion of RSV infections complicated by AOM, studies using non-otoscopy based techniques to identify AOM removed ... 15

Figure S5: Sensitivity analysis for the proportion of RSV infections complicated by AOM, studies with no reported time interval removed ... 16

Figure S6: Sensitivity analysis for the proportion of AOM-associated samples with RSV-detected, studies that didn't used PCR-based techniques removed ... 16

Figure S7: Sensitivity analysis for proportion of RSV infections complicated by AOM ,studies with quality score <75% removed ... 16

Figure S8: Sensitivity analysis for proportion of AOM-associated samples with RSV detected, studies with quality score <75% removed ... 16

Figure S9: Leave-one-out sensitivity analysis, RSV infections complicated by AOM ... 17

Figure S10: Leave-one-out sensitivity analysis, AOM-associated samples with RSV detected ... 17

Figure S11: Funnel plot for the proportion of RSV infections complicated by AOM ... 18

Figure S12: Trim and fill plot for the proportion of RSV infections complicated by AOM ... 18

Figure S13: Funnel plot for the proportion of AOM-associated samples with RSV detected ... 19

Figure S14: Trim and fill plot for the proportion of AOM-associated samples with RSV detected ... 19

Supplementary references ... 20

Material S1: PRISMA checklist

Section and Topic	Item #	Checklist item	Location where item is reported
**TITLE**			
Title	1	Identify the report as a systematic review.	
**ABSTRACT**			
Abstract	2	See the PRISMA 2020 for Abstracts checklist.	
**INTRODUCTION**			
Rationale	3	Describe the rationale for the review in the context of existing knowledge.	
Objectives	4	Provide an explicit statement of the objective(s) or question(s) the review addresses.	
**METHODS**			
Eligibility criteria	5	Specify the inclusion and exclusion criteria for the review and how studies were grouped for the syntheses.	
Information sources	6	Specify all databases, registers, websites, organisations, reference lists and other sources searched or consulted to identify studies. Specify the date when each source was last searched or consulted.	
Search strategy	7	Present the full search strategies for all databases, registers and websites, including any filters and limits used.	
Selection process	8	Specify the methods used to decide whether a study met the inclusion criteria of the review, including how many reviewers screened each record and each report retrieved, whether they worked independently, and if applicable, details of automation tools used in the process.	
Data collection process	9	Specify the methods used to collect data from reports, including how many reviewers collected data from each report, whether they worked independently, any processes for obtaining or confirming data from study investigators, and if applicable, details of automation tools used in the process.	
Data items	10a	List and define all outcomes for which data were sought. Specify whether all results that were compatible with each outcome domain in each study were sought (e.g. for all measures, time points, analyses), and if not, the methods used to decide which results to collect.	
	10b	List and define all other variables for which data were sought (e.g. participant and intervention characteristics, funding sources). Describe any assumptions made about any missing or unclear information.	
Study risk of bias assessment	11	Specify the methods used to assess risk of bias in the included studies, including details of the tool(s) used, how many reviewers assessed each study and whether they worked independently, and if applicable, details of automation tools used in the process.	
Effect measures	12	Specify for each outcome the effect measure(s) (e.g. risk ratio, mean difference) used in the synthesis or presentation of results.	
Synthesis methods	13a	Describe the processes used to decide which studies were eligible for each synthesis (e.g. tabulating the study intervention characteristics and comparing against the planned groups for each synthesis (item #5)).	
	13b	Describe any methods required to prepare the data for presentation or synthesis, such as handling of missing summary statistics, or data	

Section and Topic	Item #	Checklist item	Location where item is reported
		conversions.	
	13c	Describe any methods used to tabulate or visually display results of individual studies and syntheses.	
	13d	Describe any methods used to synthesize results and provide a rationale for the choice(s). If meta-analysis was performed, describe the model(s), method(s) to identify the presence and extent of statistical heterogeneity, and software package(s) used.	
	13e	Describe any methods used to explore possible causes of heterogeneity among study results (e.g. subgroup analysis, meta-regression).	
	13f	Describe any sensitivity analyses conducted to assess robustness of the synthesized results.	
Reporting bias assessment	14	Describe any methods used to assess risk of bias due to missing results in a synthesis (arising from reporting biases).	
Certainty assessment	15	Describe any methods used to assess certainty (or confidence) in the body of evidence for an outcome.	
**RESULTS**			
Study selection	16a	Describe the results of the search and selection process, from the number of records identified in the search to the number of studies included in the review, ideally using a flow diagram.	
	16b	Cite studies that might appear to meet the inclusion criteria, but which were excluded, and explain why they were excluded.	
Study characteristics	17	Cite each included study and present its characteristics.	
Risk of bias in studies	18	Present assessments of risk of bias for each included study.	
Results of individual studies	19	For all outcomes, present, for each study: (a) summary statistics for each group (where appropriate) and (b) an effect estimate and its precision (e.g. confidence/credible interval), ideally using structured tables or plots.	
Results of syntheses	20a	For each synthesis, briefly summarise the characteristics and risk of bias among contributing studies.	
	20b	Present results of all statistical syntheses conducted. If meta-analysis was done, present for each the summary estimate and its precision (e.g. confidence/credible interval) and measures of statistical heterogeneity. If comparing groups, describe the direction of the effect.	
	20c	Present results of all investigations of possible causes of heterogeneity among study results.	
	20d	Present results of all sensitivity analyses conducted to assess the robustness of the synthesized results.	
Reporting biases	21	Present assessments of risk of bias due to missing results (arising from reporting biases) for each synthesis assessed.	
Certainty of evidence	22	Present assessments of certainty (or confidence) in the body of evidence for each outcome assessed.	
**DISCUSSION**			
Discussion	23a	Provide a general interpretation of the results in the context of other evidence.	
	23b	Discuss any limitations of the evidence included in the review.	
	23c	Discuss any limitations of the review processes used.	
	23d	Discuss implications of the results for practice, policy, and future research.	

Section and Topic	Item #	Checklist item	Location where item is reported
**OTHER INFORMATION**			
Registration and protocol	24a	Provide registration information for the review, including register name and registration number, or state that the review was not registered.	
	24b	Indicate where the review protocol can be accessed, or state that a protocol was not prepared.	
	24c	Describe and explain any amendments to information provided at registration or in the protocol.	
Support	25	Describe sources of financial or non-financial support for the review, and the role of the funders or sponsors in the review.	
Competing interests	26	Declare any competing interests of review authors.	
Availability of data, code and other materials	27	Report which of the following are publicly available and where they can be found: template data collection forms; data extracted from included studies; data used for all analyses; analytic code; any other materials used in the review.	

From: Page MJ, McKenzie JE, Bossuyt PM, Boutron I, Hoffmann TC, Mulrow CD, et al. The PRISMA 2020 statement: an updated guideline for reporting systematic reviews. BMJ 2021;372:n71. doi: 10.1136/bmj.n71. This work is licensed under CC BY 4.0. To view a copy of this license, visit https://creativecommons.org/licenses/by/4.0/

Material S2: Search strategy

**EMBASE- OVID**	
Population	1=infant[MeSH] or infant.mp.<br>2=newborn.mp.<br>3=child[MeSH] or child.mp.<br>4=child, preschool.mp.<br>5=under 60 months.mp.<br>6=less than 5 years.mp.<br>7=1 OR 2 OR 3 OR 4 OR 5 OR 6
Condition terms- RSV	8= RSV.mp.<br>9= Human respiratory syntical virus[MeSH]<br>10= Respiratory syncytial virus infection[MeSH]<br>11= respiratory syncytial virus.mp.<br>12= bronchiolitis.mp.<br>13=8 OR 9 OR 10 OR 11 OR 12
Condition terms- AOM	14= Acute otitis media[MeSH]<br>15= Otitis media[MeSH]<br>16= AOM.mp.<br>17= Otitis[MeSH]<br>18= 14 OR 15 OR 16 OR 17
Search combination	7 AND 13 AND 18
Total number of results	844
**MEDLINE- OVID**	
Population	1= Infant [MeSH] or infant.mp.<br>2= child, preschool[MeSH]<br>3= Child [MeSH] or child.mp.<br>4=under 60 months.mp.<br>5=less than five years.mp.<br>6= 1 OR 2 OR 3 OR 4 OR 5
Condition terms- RSV	7= Respiratory Syncytial Virus Infections [MeSH]<br>8= Respiratory Syncytial Viruses [MeSH]<br>9= Respiratory Syncytial Virus, Human [MeSH]<br>10= RSV.mp.<br>11= syncytial virus.mp.<br>12= bronchiolitis.mp.<br>13= 7 OR 8 OR 9 OR 10 OR 11 OR 12
Condition terms- AOM	14= Otitis Media [MeSH]<br>15= Ear, Middle [MeSH]<br>16= Middle ear infection.mp.<br>17= Acute otitis media.mp.<br>18= Otitis.mp.<br>19= 14 OR 15 OR 16 OR 17 OR 18
Search combinations	6 AND 13 AND 19
Total number of results	186
**Global Health- OVID**	
Population	1=Infant.mp.<br>2=newborn.mp.<br>3=Child.mp.<br>4=Neonate.mp.<br>5=under 60 months.mp.<br>6=less than 5 years.mp.<br>7=1 OR 2 OR 3 OR 4 OR 5 OR 6
Condition terms- RSV	8= rsv.mp.<br>9= RSV.mp.<br>10= Respiratory syncytial virus*.mp.<br>11= bronchiolitis.mp.<br>12=8 OR 9 OR 10 OR 11

Condition terms- AOM	13=Acute otitis media.mp.<br>14= middle ear infection.mp.<br>15= AOM.mp.<br>16= Otitis.mp.<br>17= 13 OR 14 OR 15 OR 16
Search combinations	7 AND 12 AND 17
Total number of results	43

Figure S1: Analysis plan

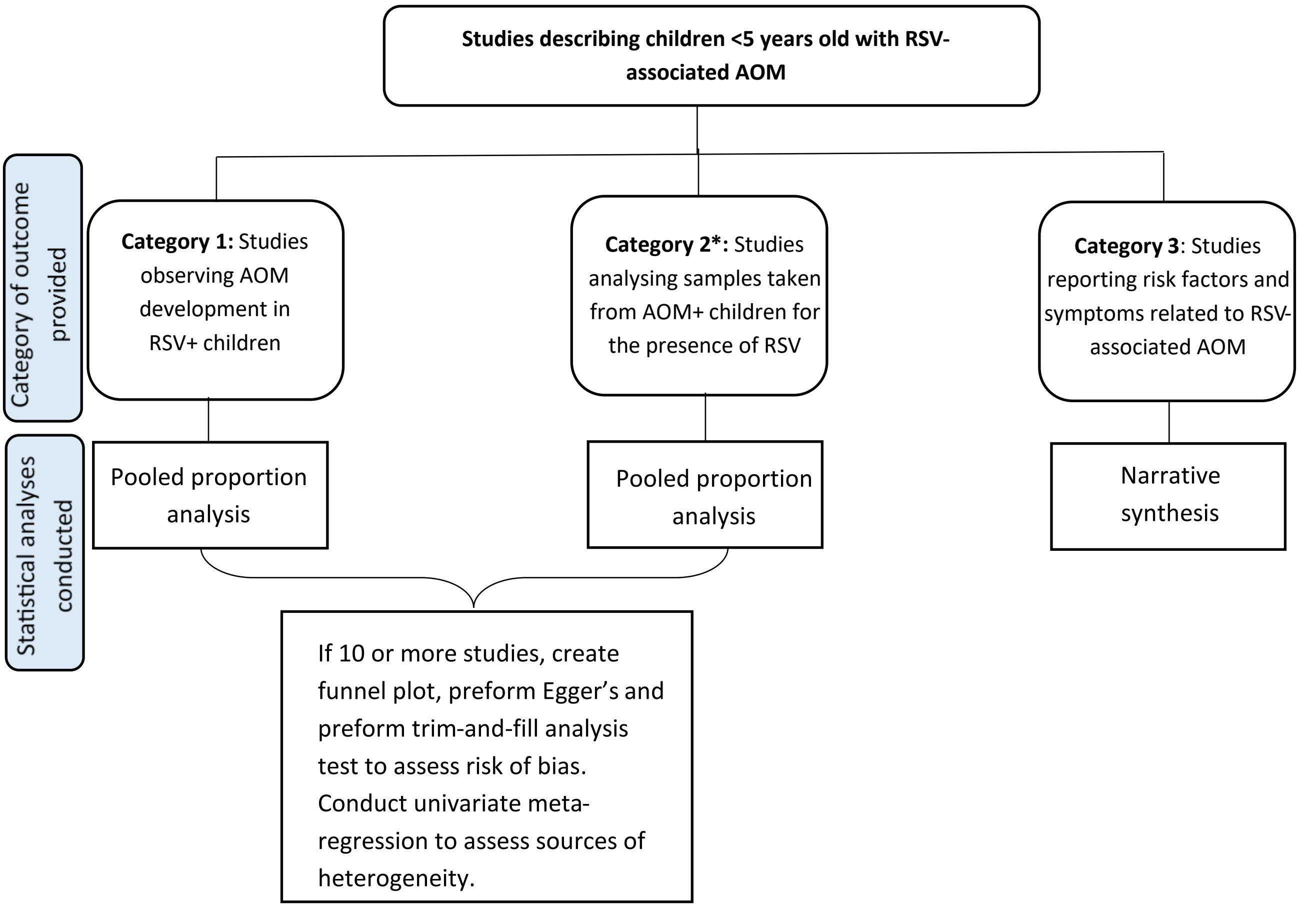

Abbreviations: RSV+= tested positive for RSV, RSV- =tested negative for RSV, AOM+ = diagnosed with acute otitis media. Studies can be in more than one category.

* *Kleemola et al. 2006* was excluded from all but one Category 2 pooled proportion analysis, due to containing an overlapping patient population with *Nokso-Koivisto et al.2004*. However, its unique RSV-bacterial co-infection data meant it was included within the RSV-bacterial co-infection analysis (Figure 3D). It was also included in publication bias tests as a minimum of 10 studies was required to perform analyses.

Material S3: Proportion calculations

Calculation for the proportion of RSV infections complicated by AOM:

Numerator = Number of RSV infections associated with a complicating diagnosis of AOM

Denominator = Number of RSV infections

Calculation of the proportion of AOM-associated samples with RSV detected:

Numerator = Number of AOM-associated samples (samples taken from children diagnosed with AOM) with RSV detected. For studies utilising multiple sampling sites, this figure reflects the combined total of all nasopharyngeal and middle ear fluid samples with RSV detected.

Denominator = Number of AOM-associated samples collected. For studies utilising multiple sampling sites, this figure reflects the combined total of all nasopharyngeal and middle ear fluid samples taken.

Both proportions were logit transformed and calculated using random-effects modelling in R

Material S4: Manual odds ratio calculation

	No. of children diagnosed with AOM	No. of children not diagnosed with AOM
No. of RSV positive children with risk factor present	**A**	**B**
No. of RSV positive children with risk factor not present	**C**	**D**

Non-adjusted odds ratio were calculated using the standard formula= (a/b)/(c/d). No continuity corrections were used as all cells had non-zero counts.

Material S5: Quality assessment

To assess risk of bias, Joanna Briggs Institute (JBI) Critical Appraisal Tools were used for cohort studies, cross-sectional studies, case control studies or quasi-experimental studies depending on the study designs.

Cohort Study quality assessment:

Q1: Were the two groups similar and recruited from the same population? **Q2**: Were the exposures measured similarly to assign people to both exposed and unexposed groups? **Q3**: Was the exposure measured in a valid and reliable way? **Q4**: Were confounding factors identified? **Q5**: Were strategies to deal with confounding factors stated? **Q6:** Were the groups/participants free of the outcome at the start of the study (or at the moment of exposure)? **Q7:** Were the outcomes measured in a valid and reliable way? **Q8**: Was the follow up time reported and sufficient to be long enough for outcomes to occur? **Q9:** Was follow up complete, and if not, were the reasons to loss to follow up described and explored? **Q10:** Were strategies to address incomplete follow up utilized? **Q11:** Was appropriate statistical analysis used?

Author	Q1	Q2	Q3	Q4	Q5	Q6	Q7	Q8	Q9	Q10	Q11	Score	Percentage
Bulut et. al. (2007) [1]	NA	NA	Yes	No	No	Yes	Yes	Yes	Yes	NA	Yes	6/8	75%
Chonmaitree et. al. (2008) [2]	NA	NA	Yes	No	No	Yes	Yes	Yes	No	No	Yes	7/9	78%
Gomaa et. al. (2012) [3]	NA	NA	Yes	No	No	Unclear	Yes	Yes	Unclear	No	Yes	5/9	56%
Heikkinen et. al. (2017)[4]	NA	NA	Yes	No	No	Unclear	Yes	Yes	No	No	Yes	6/9	67%
Kleemola et. al. (2006) [5]	NA	NA	Yes	No	No	Yes	Yes	Yes	Yes	NA	Yes	5/9	56%
Nokso-Koivisto et. al. (2012) [6]	NA	NA	Yes	No	No	Yes	Yes	Yes	Yes	NA	Yes	5/9	56%
Nokso-Koivisto et. al. (2004) [7]	NA	NA	Yes	No	No	Yes	Yes	Yes	No	No	Yes	5/9	56%
Pettigrew et. al. (2011) [8]	NA	NA	Yes	Yes	Yes	Yes	Yes	Yes	Yes	NA	Yes	8/9	82%
Ruohola et. al. (2013) [9]	Yes	Yes	Yes	No	Yes	Unclear	Yes	Yes	Unclear	No	Yes	9/11	82%
Sagai et. al. (2004) [10]	NA	NA	Yes	No	No	Unclear	Yes	Yes	Unclear	No	Yes	5/9	56%
Thomas et. al. (2021) [11]	NA	NA	Yes	No	No	Unclear	Yes	Yes	Unclear	No	Yes	7/9	78%
Toivonen et. al. (2020) [12]	Yes	Yes	Yes	No	No	Yes	Yes	Yes	No	No	Yes	7/11	64%
Wrotek et. al. (2020) [13]	Yes	Yes	Yes	No	No	Yes	Yes	Yes	Unclear	No	Yes	7/11	64%

Cross-sectional study quality assessment:

Q1: Were the criteria for inclusion in the sample clearly defined? **Q2:** Were the study subjects and the setting described in detail? **Q3:** Was the exposure measured in a valid and reliable way? **Q4:** Were objective, standard criteria used for measurement of the condition? **Q5**: Were confounding factors identified? **Q6:** Were strategies to deal with confounding factors stated? **Q7:** Were the outcomes measured in a valid and reliable way? **Q8:** Was appropriate statistical analysis used?

Author	Q1	Q2	Q3	Q4	Q5	Q6	Q7	Q8	Score	Percentage
Adar et. al. (2025) [14]	Yes	Yes	Yes	Yes	No	Yes	Yes	Yes	7/8	88%
Kuczborska et. al. (2021) [15]	Yes	Yes	Yes	Yes	No	No	Unclear	Yes	5/8	63%
Majadla et. al. (2025)[16]	Yes	Yes	Yes	Yes	No	No	Yes	Yes	6/8	75%
Marom et. al. (2019) [17]	Yes	Yes	Yes	Yes	No	No	Yes	Yes	6/8	75%
Marom et. al. (2017) [18]	Yes	Yes	Yes	Yes	No	No	Yes	Yes	4/8	50%
Papan et. al. (2020) [19]	Yes	Yes	Yes	Yes	No	No	Unclear	Yes	7/8	88%
Sawada et. al. (2020) [20]	Yes	Yes	Yes	Yes	No	No	Yes	Yes	6/8	75%

Case-control study assessment:

Q1: Were the groups comparable other than the presence of disease in cases or the absence of disease in controls? **Q2:** Were cases and controls matched appropriately? **Q3:** Were the same criteria used for identification of cases and controls? **Q4:** Was exposure measured in a standard, valid and reliable way? **Q5:** Was exposure measured in the same way for cases and controls? **Q6**: Were confounding factors identified? **Q7**: Were strategies to deal with confounding factors stated? **Q8:** Were outcomes assessed in a standard, valid and reliable way for cases and controls? **Q9:** Was the exposure period of interest long enough to be meaningful? **Q10:** Was appropriate statistical analysis used?

Author	Q1	Q2	Q3	Q4	Q5	Q6	Q7	Q8	Q9	Q10	Score	Percentage
Tomochika et. al. (2009) [21]	Yes	No	Yes	Yes	Yes	No	No	Yes	Yes	Yes	7/10	70%

Quasi-experimental study assessment:

Q1: Is it clear in the study what is the "cause" and what is the "effect" (i.e. there is no confusion about which variable comes first)? **Q2:** Was there a control group? **Q3:** Were participants included in any comparisons similar? **Q4:** Were the participants included in any comparisons receiving similar treatment/care, other than the exposure or intervention of interest? **Q5:** Were there multiple measurements of the outcome, both pre and post the intervention/exposure? **Q6:** Were the outcomes of participants included in any comparisons measured in the same way? **Q7:** Were outcomes measured in a reliable way? **Q8:** Was follow-up complete and if not, were differences between groups in terms of their follow-up adequately described and analyzed? **Q9:** Was appropriate statistical analysis used?

Author	Q1	Q2	Q3	Q4	Q5	Q6	Q7	Q8	Q9	Score	Percentage
Alonso et. al. (2007) [22]	Yes	No	Yes	Yes	NA	Yes	Yes	NA	Yes	5/6	83%

Table S1: Study design and population demographics

**Author**	**Study type**	**Main study population**	**Study data type provided**	**Overall outcome measured**	**Publication date**	**Study period**	**Country**	**Care setting**	**Cohort size**	**Age range (months)**	**Mean age (months)**	**Median age**	**Gender distribution (percentage male)**	**Ethnicity/ race**	**Breast-feeding status**	**Cigarette exposure**	**Preterm birth (gestational age < 37 weeks)**	**Method of diagnosing AOM (i.e symptom-based, otoscopic exam)**	**Method for RSV detection**
Adar et al. (2025)	Cross-sectional	RSV+	RD	Effect of CRP levels on bronchiolitis outcomes	2025	Jan2018 to Mar2022	Israel	Tertiary	1874	0 to 24	6.7	NA	55.90%	Bedouin 62.6%, Jewish 37.4%	NA	NA	62 (3.3%)	ICD-9 codes	RT-PCR
Alonso et al. (2007)	Time-series analysis	RSV+	RD	Seasonality of Bronchiolitis	2007	Jan1992 to Dec2004	Spain	Secondary	1324	0 to 24	6.89	NA	63.80%	NA	NA	NA	NA	NA	Immunofluorescent assay
Bulut et al. (2007)	Prospective cohort	AOM +	SD	AOM-pathogen associations	2006	Mar2003 to Dec2004	Turkey	Secondary	120	6 to 144 (s data for 6-24)	NA	32.58	60.00%	NA	NA	NA	NA	Otoscopy: Presence of MEF plus tm changes	RT-PCR
Chonmaitree et al. (2008)	Prospective cohort	Healthy-monitored for RSV	RD + ORD	Respiratory infections in AOM	2008	Jan2003 to Mar2006	USA	Primary	294	6 to 36	13.7	12	51.00%	White 59%,Black 31%, Biracial 8%,Asian 2%, Hispanic/Latino 56%	47% breast-fed	35%	NA	Otoscopy: MEF presence, tm inflammation Acute symptoms	RT-PCR and EIA
Gomaa et al. (2012)	Prospective cohort study	AOM and concurrent bronchiolitis	SD	AOM features in children with bronchiolitis	2011	winter to spring 2009	Egypt	Secondary	180	3 to 18	7.45	NA	55.56%	NA	NA	NA	NA	Otoscopy: Ear discharge, abnormal tm + signs of inflammation	ELISA

Heikkinen et al. (2017)	Prospective cohort	Healthy-monitored for RSV	RD + ORD	RSV-related complications	2017	Oct2000 to May 2002	Finland	Primary	2231	<6 to 156 (s data for <24)	NA	NA	51.60%	NA	NA	35.70%	9.14%	Otoscopy, tympanometry, acoustic reflectometry	RT-PCR
Kleemola et al. (2006)	Prospective cohort	Healthy children	SD	AOM co-infections	2006	1994 to 1999	Finland	Secondary	353	2 to 24	NA	NA	NA	NA	NA	NA	NA	Otoscopy and symptoms	RT-PCR, TR-FIA
Kuczborska et al. (2021)	Cross-sectional	Admitted with respiratory tract infections	RD + ORD	Clinical features of RSV-hospitalised infants	2020	Jan2018 to Mar2020	Poland	Secondary	448	<18	NA	RSV+=3 RSV-=6	60.04%	NA	NA	NA	NA	NA	Rapid-VIDITEST
Majadla et al. (2025)	Cross-sectional	Admitted with respiratory infections + concurrent AOM	SD	Epidemiology of viruses and AOM	2025	Mar2017 to Feb2022	Israel	Secondary	376	1-73.8 (S data for <24)	NA	17.6	61.20%	NA	NA	NA	NA	ICD-9 codes	PCR
Marom et al. (2019)	Cross-sectional	Admitted with respiratory infections + concurrent AOM	SD	Viral associations with AOM	2019	Oct2012 to Apr2017	Israel	Secondary	249	0-72 (s data for <12)	NA	15	62.00%	NA	NA	NA	NA	ICD-9 codes	Antigen assays, PCR
Marom et al. (2017)	Cross-sectional	AOM +	SD	Pneumococcal vaccine impact on AOM	2017	Jan2010 to Dec2015	Israel	Secondary	409	<12	NA	NA	62.00%	NA	23% breast-fed	NA	NA	ICD-9 codes	Respiratory panels
Nokso-Koivisto et al. (2012)	Propsective cohort	Healthy	SD	Viral associations with AOM	2011	Jan2003 to	USA	Secondary	200	6 to 36	NA	NA	51.00%	White 59.5%, Black 28%, Asian 3%,	53% breast-fed	31%	NA	Otoscopy: MEF presence, TM inflammation, acute symptoms	Enzyme immunoassay, qPCR

						Mar2007								Biracial 9.5%, Hispanic 45%, Non-Hispanic 55%					
Nokso-Koivisto et al. (2004)	Prospective cohort	Healthy	SD	AOM epidemiology and risk factors	2003	Cohort: Apr1994 to Jul1997, Vacc: Dec1995 to Mar1999	Finland	Secondary	Total: 940, Cohort: 329, Vacc: 611	2 to 24	NA	NA	Total: 50.4%, Cohort: 48%, Vacc: 52%	NA	NA	NA	NA	Otoscopy: Abnormal tm, acute symptoms	RT-PCR, TR-FIA
Papan et al. (2020)	Cross-sectional	*RSV +	RD	Antibiotic use in respiratory infections	2020	Apr2014 to Apr2018	Germany	Secondary	573	<24	NA	5	56.70%	NA	NA	NA	NA	NA	TR-FIA, multiplex PCR
Pettigrew et al. (2011)	Prospective cohort	Healthy-monitored for RSV	RD + ORD	AOM-pathogen associations	2011	Jan2003 to Mar2007	USA	Secondary	194	6 to 36	14.2	NA	51.00%	White 20%, Black 29%, 43%, Other 8%	34% breast-fed	27%	NA	Otoscopy: Sign of acute infection, TM inflammation, MEF presence	qPCR
Ruohola et al. (2013)	Prospective cohort study	AOM + (case), AOM- (control)	SD	AOM-pathogen associations	2013	2006 to 2009	Finland	Secondary	Total: 505<br><br>Non-AOM group: 187<br><br>AOM group: 318	6 to 35	16	NA	Case=54% case=57%	NA	breast fed: 98% control, 96% case	24% control, 33% case	NA	Acute symptoms Otoscopy: MEF presence, abnormal tm	Fluroimmunoassays, RT-qPCR, multiplex PCR

Sagai et al. (2004)	Prospective cohort	RSV+	RD + ORD	RSV's association with AOM	2004	Nov2001 to Oct2002	Japan	Secondary	230	1 to 108 (s data for <24)	28.8	NA	NA	NA	NA	NA	NA	Otoscopy: MEF presence and tm inflammation	Enzyme immunoassay
Sawada et al. (2020)	Cross-sectional	AOM +	SD	AOM-pathogen associations	2020	Jan2016 to Dec2017	Japan	Primary	122	4 to 36	NA	14.5	NA	NA	NA	NA	NA	Symptom-based and pneumatic otoscopy	
Thomas et al. (2021)	Prospective cohort	Healthy-monitored for RSV	RD	RSV-complications	2021	Sep2017 to Jun2018	Finland	Primary	408	0 to 3	NA	NA	51.00%	NA	NA	77 (18.9%)	27 (6.6%)	Otoscopy: MEF presence, TM inflammation, signs of acute infection	RT-PCR
Toivonen et al. (2020)	Prospective cohort	Healthy-monitored for RSV	RD + ORD	Characteristics of RSV infection	2020	Jan2008 to Apr2010	Finland	Primary	923	0-24	NA	RSV+ = 9.8<br><br>RSV- = 11.3	52.90%	NA	60.3% breast-fed	5.5% maternal, 17,2% paternal	NA	Pneumatic otoscopy and tympanometry	RT-PCR
Tomochika et al. (2009)	Retrospective case-control	RSV+ AOM + (case), RSV+ AOM- (controls)	RD + ORD	Factors linked to RSV-associated AOM	2009	Jan2004 to Dec2007	Japan	Secondary	Total: 148 case: 46 control:102	6 to 35	case=15.8 control=14.1	NA	case=58.7% control=50%	NA	NA	NA	NA	Otoscopy: Inflamed tm and MEF presence	RT-PCR and antigen tests
Wrotek et al. (2020)	Prospective cohort study	RSV+	RD + ORD	RSV-complications	2020	2017 to 2018	Poland	Tertiary	111	0 to 22	NA	3	NA	NA	NA	NA	NA	Otoscopy	NA

Abbreviations for Supplementary Material 4: RSV+ = tested positive for respiratory syncytial virus (RSV), RSV- = tested negative for RSV, AOM+ = has confirmed diagnosis of acute otitis media (AOM), AOM- = does not have confirmed diagnosis of AOM, S data = stratified data, tm = tympanic membrane, SD = provides sample data, RD = provides RSV-complication data, ORD = provides odds ratio data, MEF = middle ear fluid, Vacc = vaccination trial, Cohort = cohort study, Rapid-VIDITEST = Rapid-Viral Detection Test, TRFIA = Time-Resolved Fluorescence Immunoassay, EIA = Enzyme Immunoassay, ELISA = Enzyme-Linked Immunosorbent Assay, RT-PCR = Reverse Transcription Polymerase Chain Reaction, qPCR = Quantitative Polymerase Chain Reaction, RT-qPCR = Reverse Transcription Quantitative Polymerase Chain Reaction

Table S2: Surveillance time-window data

Author	Maximum time-window between RSV infection and associated AOM diagnosis (days)
Heikkinen et al. (2017)	14
Thomas et al. (2021)	14
Wrotek et al. (2020)	7
Pettigrew et al. (2011)	17
Adar et al. (2025)	NA
Toivonen et al. (2020)	14
Sagai et al. (2004)	28
Chonmaitree et al. (2008)	28
Tomochika et al. (2009)	NA
Papan et al. (2020)	NA
Kuczborska et al. (2021)	NA
Alonso et al. (2007)	NA

Table S3: Meta Regression: The proportion of RSV infections complicated by AOM

	estimate	SE	95% CI	P-value
**Year published**	-0.0985	0.0740	-0.2435; 0.0465	0.1830
**Maximum age (months) of child included**	0.0153	0. 0116	-0.0074; 0.0381	0.1873
**Maximum time window (days) between RSV-positive result and AOM diagnosis**	0.0235	0.0404	0.0557; 0.1027	0.5611
**Intrcpt (Record-based)**	-2.5468	0.4934	-3.5140; -1.5797	<.0001
**Prospective (active surveillance)**	2.7237	0.6435	1.4625; 3.9848	<.0001
**Intrcpt (hospital)**	-1.5222	0.5732	-2.6457; -0.3987	0.0079
**Outpatient**	1.6818	0.9913	-0.2611; 3.6247	0.0898
**Intrcpt (assay)**	-1.3555	0.9110	-3.1409; 0.4300	0.1368
**PCR**	0.5869	1.1135	-1.5955; 2.7694	0.5981

Table S4: Meta Regression: The proportion of AOM-associated samples with RSV-detected

	estimate	SE	95% CI	P-value
**Maximum age (months) of child included**	0.0026	0.0082	-0.0134; 0.0186	0.7504
**Intrcpt (Cross-sectional)**	-0.9492	0.4559	-1.8428; -0.0557	0.0373
**Prospective**	-0.4058	0.6127	-1.6065; 0.7950	0.5078
**Intrcpt (ICD-9)**	-0.8291	0.5197	-1.8477; 0.1896	0.1107
**Otoscopy**	-0.5179	0.6367	-1.7659; 0.73	0.4160
**Intrcpt (assay)**	-0.4480	0.5940	-1.6123; 0.7162	0.4507
**PCR**	-0.9324	0.6727	-2.2509; 0.3861	0.1658
**Year published**	0.0429	0.0443	-0.0439; 0.1297	0.3329

Figure S2: Subgroups for the proportion of RSV infections complicated by AOM

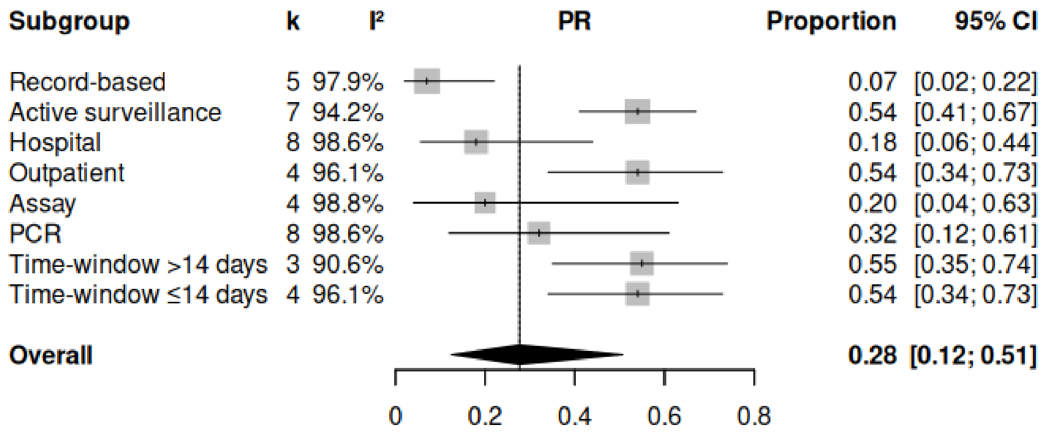

Figure S3: Subgroups for the proportion of AOM-associated samples with RSV detected

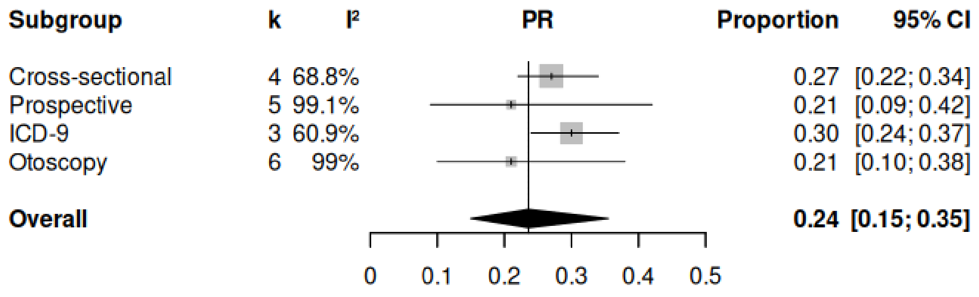

Figure S4: Sensitivity analysis for the proportion of RSV infections complicated by AOM, studies using non-otoscopy based techniques to identify AOM removed

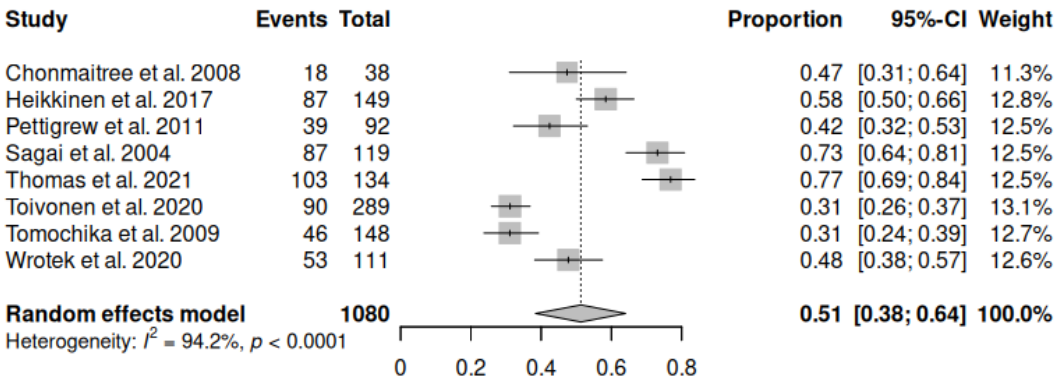

Figure S5: Sensitivity analysis for the proportion of RSV infections complicated by AOM, studies with no reported time interval removed

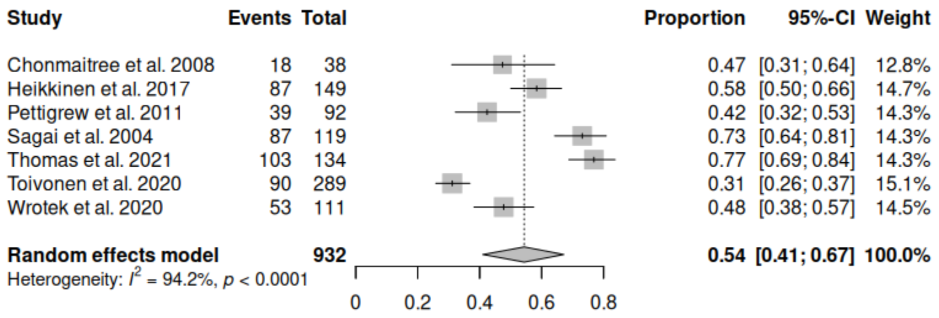

Figure S6: Sensitivity analysis for the proportion of AOM-associated samples with RSV-detected, studies that didn't used PCR-based techniques removed

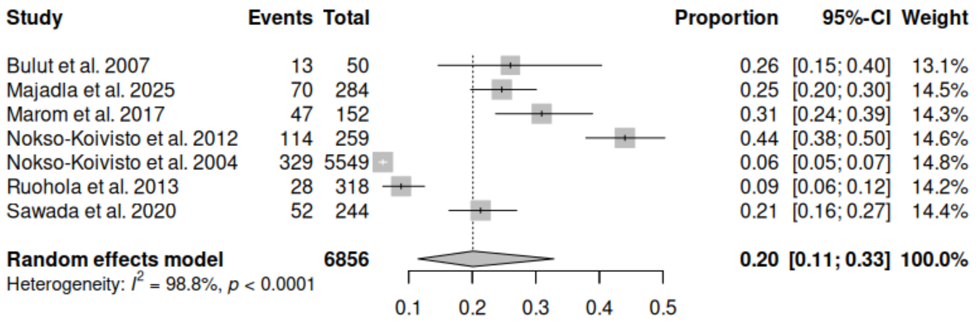

Figure S7: Sensitivity analysis for proportion of RSV infections complicated by AOM ,studies with quality score <75% removed

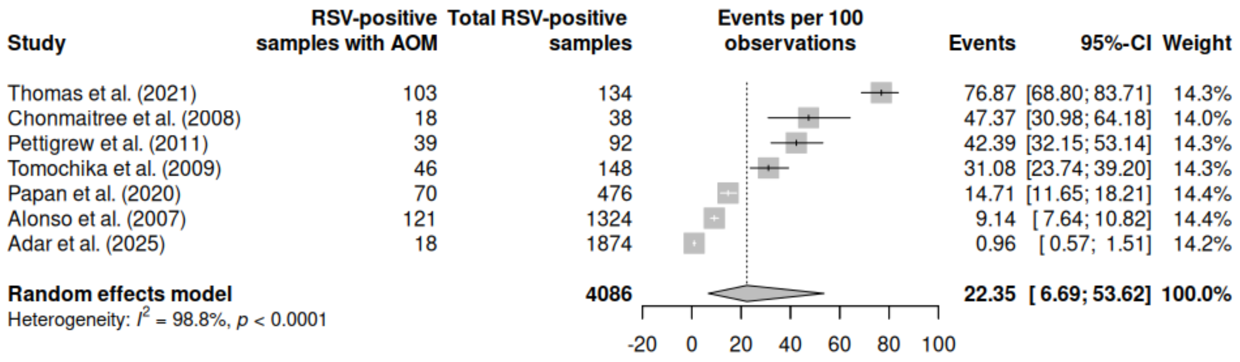

Figure S8: Sensitivity analysis for proportion of AOM-associated samples with RSV detected, studies with quality score <75% removed

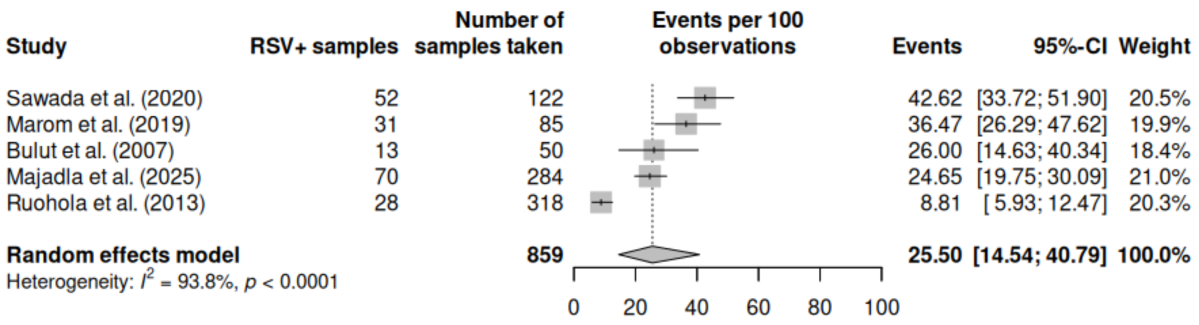

Figure S9: Leave-one-out sensitivity analysis, RSV infections complicated by AOM

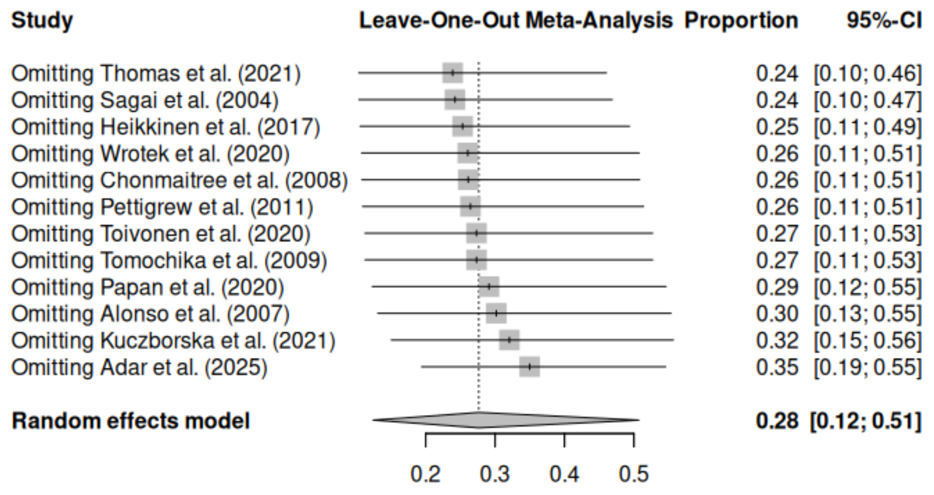

Figure S10: Leave-one-out sensitivity analysis, AOM-associated samples with RSV detected

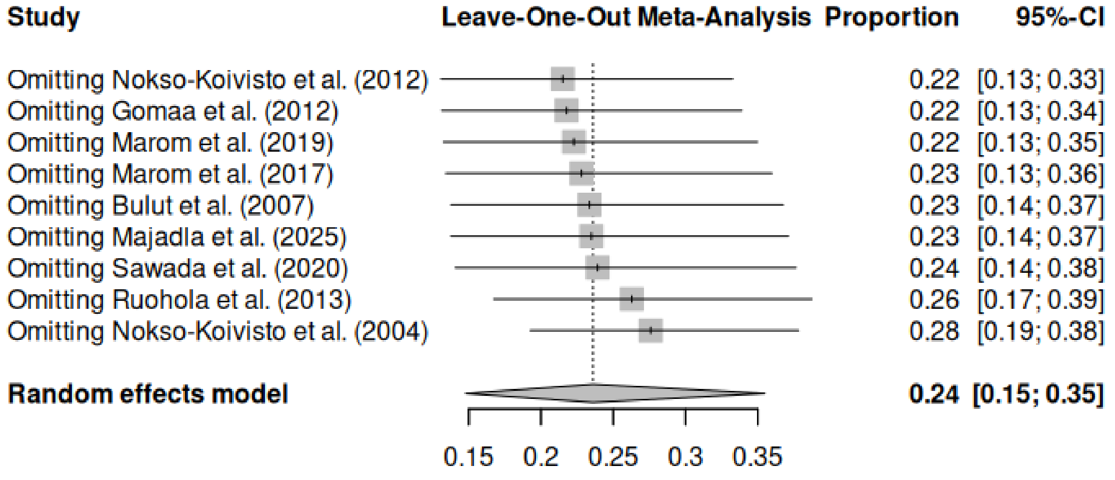

Figure S11: Funnel plot for the proportion of RSV infections complicated by AOM

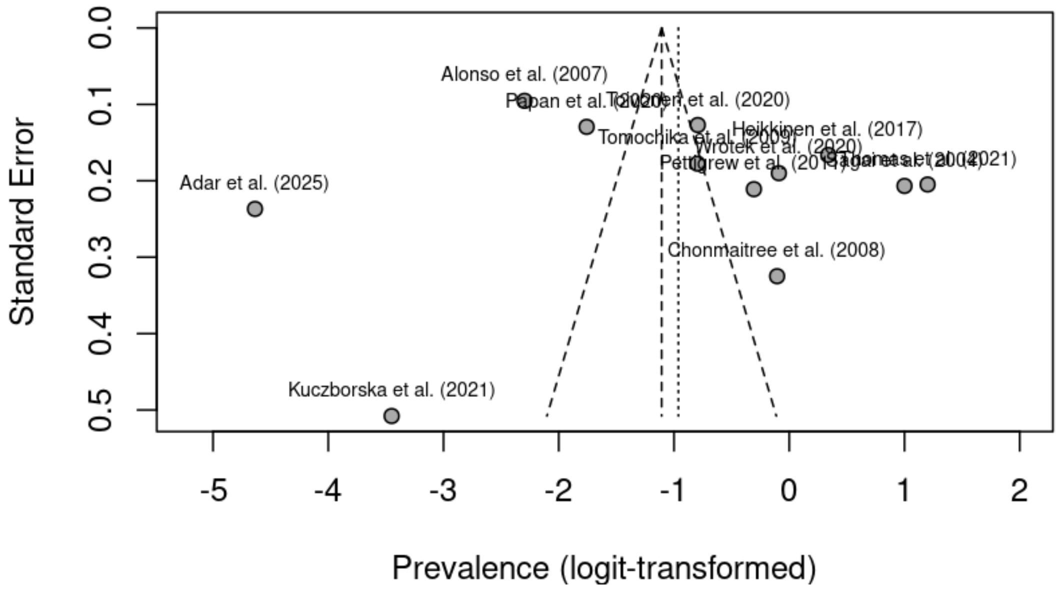

Figure S12: Trim and fill plot for the proportion of RSV infections complicated by AOM

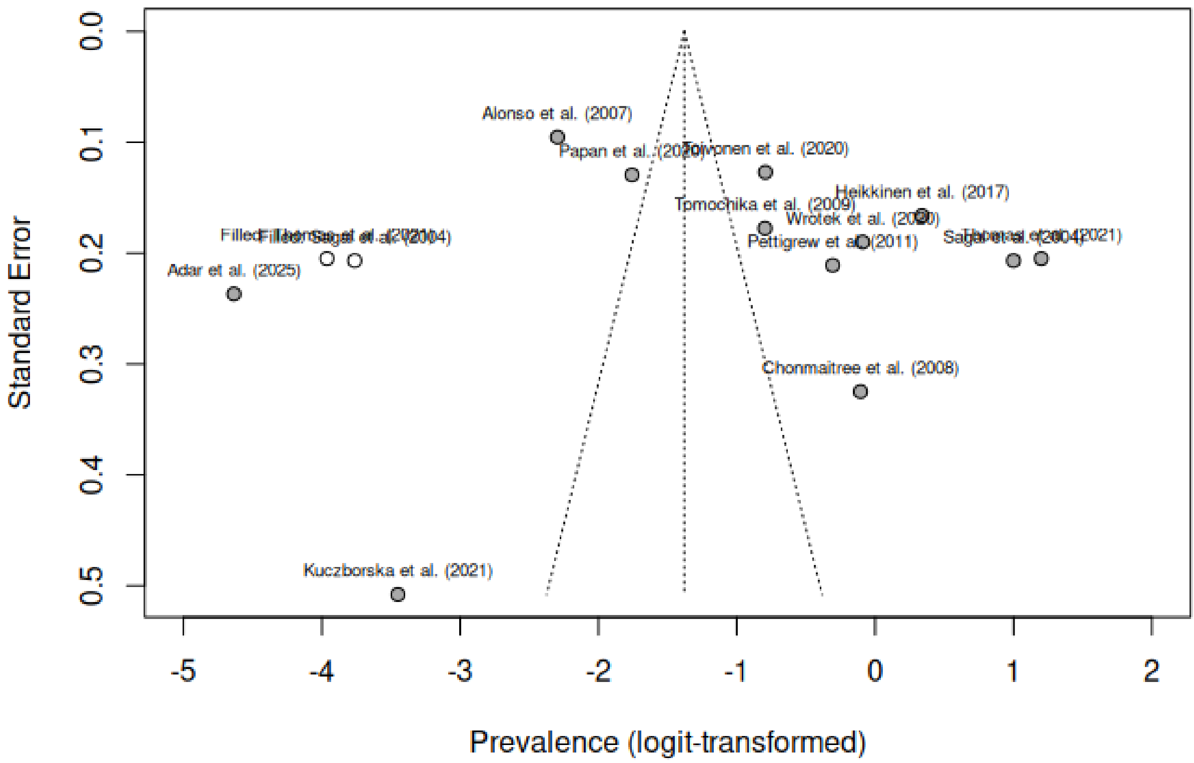

Figure S13: Funnel plot for the proportion of AOM-associated samples with RSV detected

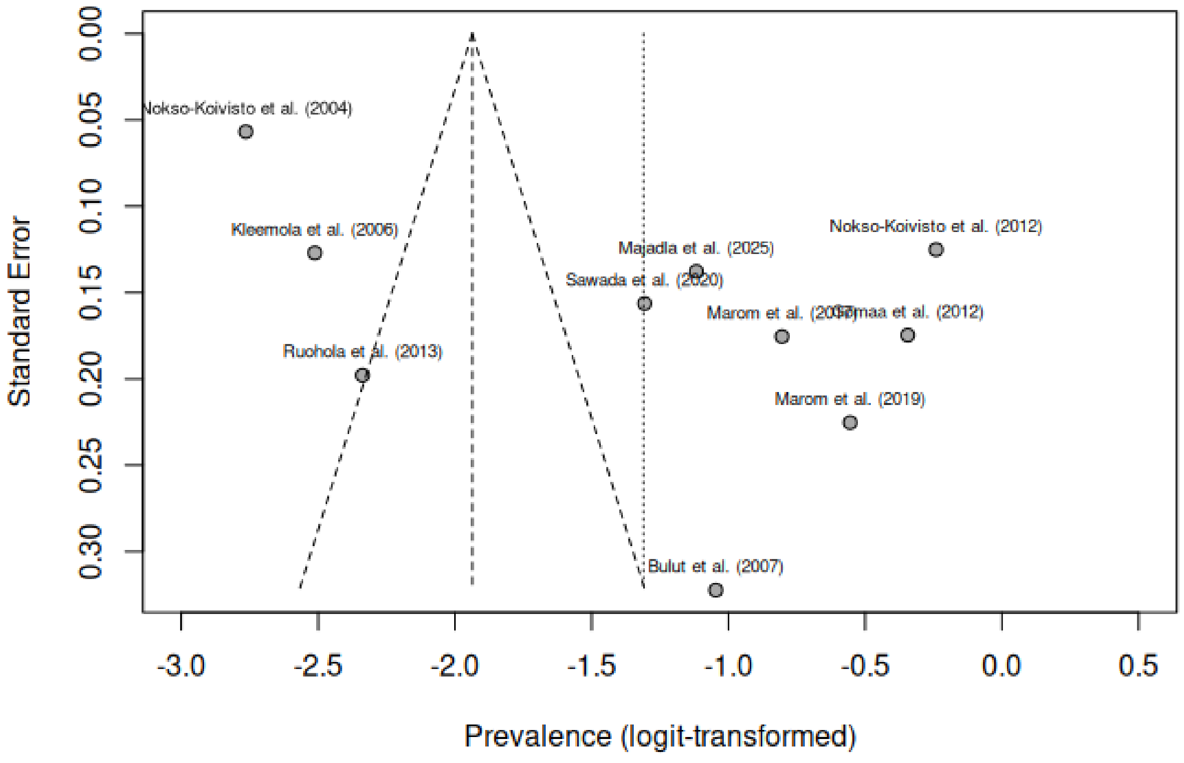

Figure S14: Trim and fill plot for the proportion of AOM-associated samples with RSV detected

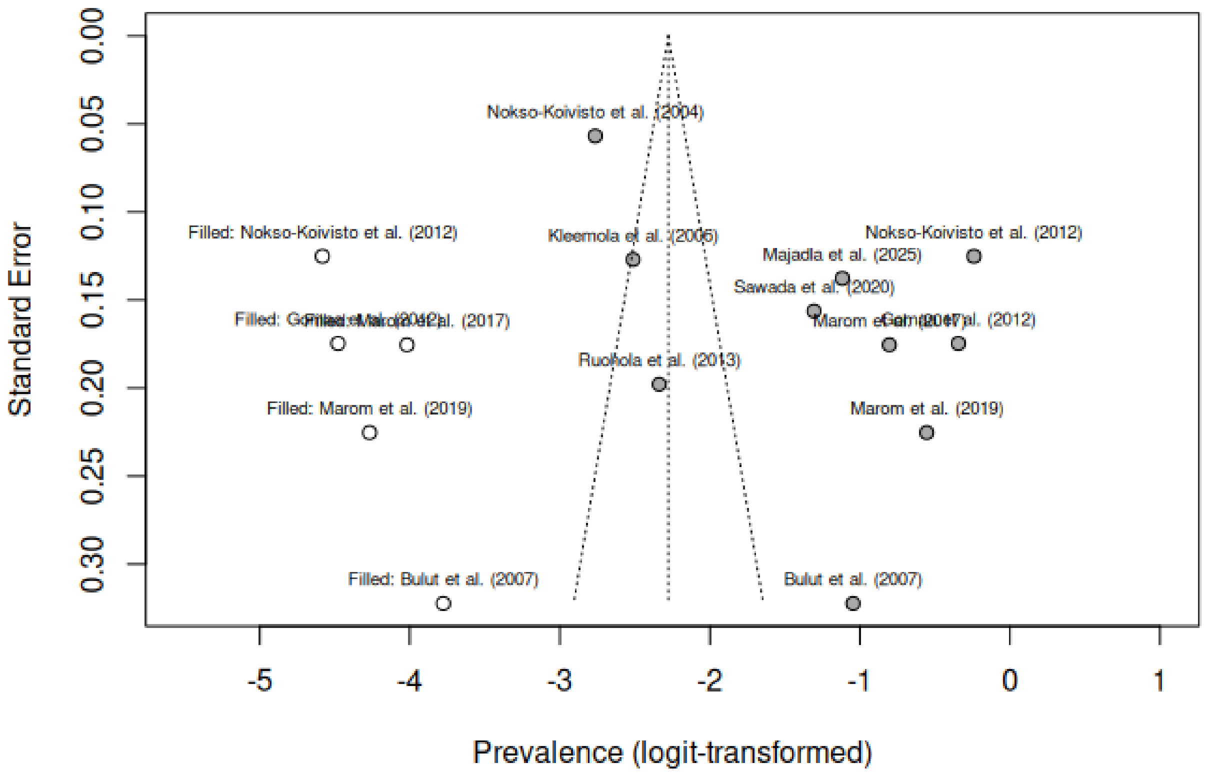

Kleemola et al. 2006 Included for forest plots as 10 studies required for publication bias tests

Supplementary references

1 Bulut Y, Güven M, Otlu B, Yenişehirli G, Aladağ I, Eyibilen A, et al. Acute otitis media and respiratory viruses. Eur J Pediatr. 2007;166:223–8.

2 Chonmaitree T, Revai K, Grady JJ, Clos A, Patel JA, Nair S, et al. Viral upper respiratory tract infection and otitis media complication in young children. Clinical Infectious Diseases. 2008;46:815–23.

3 Gomaa MA, Galal O, Mahmoud MS. Risk of acute otitis media in relation to acute bronchiolitis in children. Int J Pediatr Otorhinolaryngol. 2012;76:49–51.

4 Heikkinen T, Ojala E, Waris M. Clinical and Socioeconomic Burden of Respiratory Syncytial Virus Infection in Children. J Infect Dis. 2017;215:17–23.

5 Kleemola M, Nokso-Koivisto J, Herva E, Syrjänen R, Lahdenkari M, Kilpi T, et al. Is there any specific association between respiratory viruses and bacteria in acute otitis media of young children? Journal of Infection. 2006;52:181–7.

6 Nokso-Koivisto J, Pyles RB, Miller AL, Patel JA, Loeffelholz M, Chonmaitree T. Viral Load and Acute Otitis Media Development after Human Metapneumovirus Upper Respiratory Tract Infection. Pediatr Infect Dis J. 2012;31:763–6.

7 Nokso-Koivisto J, Räty R, Blomqvist S, Kleemola M, Syrjänen R, Pitkäranta A, et al. Presence of Specific Viruses in the Middle Ear Fluids and Respiratory Secretions of Young Children with Acute Otitis Media. J Med Virol. 2004;72:241–8.

8 Pettigrew MM, Gent JF, Pyles RB, Miller AL, Nokso-Koivisto J, Chonmaitree T. Viral-bacterial interactions and risk of acute otitis media complicating upper respiratory tract infection. J Clin Microbiol. 2011;49:3750–5. Medline:21900518

9 Ruohola A, Pettigrew MM, Lindholm L, Jalava J, Räisänen KS, Vainionpää R, et al. Bacterial and viral interactions within the nasopharynx contribute to the risk of acute otitis media. Journal of Infection. 2013;66:247–54.

10 Sagai S, Suetake M, Yano H, Yoshida M, Ohyama K, Endo H, et al. Relationship between respiratory syncytial virus infection and acute otitis media in children. Auris Nasus Larynx. 2004;31:341–5.

11 Thomas E, Mattila JM, Lehtinen P, Vuorinen T, Waris M, Heikkinen T. Burden of Respiratory Syncytial Virus Infection During the First Year of Life. J Infect Dis. 2021;223:811–7.

12 Toivonen L, Karppinen S, Schuez-Havupalo L, Teros-Jaakkola T, Mertsola J, Waris M, et al. Respiratory syncytial virus infections in children 0–24 months of age in the community. Journal of Infection. 2020;80:69–75.

13 Wrotek A, Kobiałka M, Grochowski B, Kamińska I, Pędziwiatr K, Skoczek-Wojciechowska A, et al. Respiratory Complications in Children Hospitalized with Respiratory Syncytial Virus Infection. Adv Exp Med Biol. 2020;1279:113–20.

14 Adar A, Goldbart AD, Burrack N, Geva N, Cohen B, Golan-Tripto I. C-Reactive Protein Is Associated with Severity in Hospitalized Children with Respiratory Syncytial Virus Bronchiolitis. Isr Med Assoc J. 2025;27:165–71.

15 Kuczborska K, Rustecka A, Wawrzyniak A, Będzichowska A, Kalicki B. Manifestations and risk factors in children hospitalized with respiratory syncytial virus infection. Arch Pediatr Infect Dis. 2021;9 e108723..

16 Majadla O, Fellner A, Mizrakli Y, Hirschfeld Z, Muallem-Kalmovich L, Gavriel H, et al. Epidemiology of respiratory viruses in pediatric acute otitis media during the COVID-19 pandemic: a cross-sectional study. Discover public health. 2025;22:1–8.

17 Marom T, Nokso-Koivisto J, Chonmaitree T. Viral–Bacterial Interactions in Acute Otitis Media. Curr Allergy Asthma Rep. 2012;12:551–8.

18 Marom T, Israel O, Gavriel H, Pitaro J, Baker AA, Eviatar E. Comparison of first year of life acute otitis media admissions before and after the 13-valent pneumococcal conjugate vaccine. Int J Pediatr Otorhinolaryngol. 2017;97:251–6.

19 Papan C, Willersinn M, Weiß C, Karremann M, Schroten H, Tenenbaum T. Antibiotic utilization in hospitalized children under 2 years of age with influenza or respiratory syncytial virus infection - A comparative, retrospective analysis. BMC Infect Dis. 2020;20:606.

20 Sawada S, Okutani F, Kobayashi T. Comprehensive Detection of Respiratory Bacterial and Viral Pathogens in the Middle Ear Fluid and Nasopharynx of Pediatric Patients With Acute Otitis Media. Pediatr Infect Dis J. 2019;38:1199–203.

21 Tomochika K, Ichiyama T, Shimogori H, Sugahara K, Yamashita H, Furukawa S. Clinical characteristics of respiratory syncytial virus infection-associated acute otitis media. Pediatrics International. 2009;51:484–7.

22 Alonso A, Andres JM, Garmendia JR, Diez I, Gil JM, Ardura J. Bronchiolitis due to respiratory syncytial virus in hospitalized children: A study of seasonal rhythm. Acta Paediatrica, International Journal of Paediatrics. 2007;96:731–5.
